# Probing an AI regression model for hand bone age determination using gradient-based saliency mapping

**DOI:** 10.1038/s41598-021-90157-y

**Published:** 2021-05-19

**Authors:** Zhiyue J. Wang

**Affiliations:** Department of Radiology, Children’s Health and University of Texas Southwestern Medical Center, 1935 Medical District Drive, F1-02, Dallas, TX 75235 USA

**Keywords:** Medical research, Anatomy, Musculoskeletal system

## Abstract

Understanding how a neural network makes decisions holds significant value for users. For this reason, gradient-based saliency mapping was tested on an artificial intelligence (AI) regression model for determining hand bone age from X-ray radiographs. The partial derivative (PD) of the inferred age with respect to input image intensity at each pixel served as a saliency marker to find sensitive areas contributing to the outcome. The mean of the absolute PD values was calculated for five anatomical regions of interest, and one hundred test images were evaluated with this procedure. The PD maps suggested that the AI model employed a holistic approach in determining hand bone age, with the wrist area being the most important at early ages. However, this importance decreased with increasing age. The middle section of the metacarpal bones was the least important area for bone age determination. The muscular region between the first and second metacarpal bones also exhibited high PD values but contained no bone age information, suggesting a region of vulnerability in age determination. An end-to-end gradient-based saliency map can be obtained from a black box regression AI model and provide insight into how the model makes decisions.

## Introduction

As artificial intelligence (AI) and machine learning models are becoming more prevalent in radiology for a variety of applications, it is important to understand how these models make decisions^1,2^. Insight into these models helps build trust in the software, enables the user to obtain new knowledge, and is useful for quality assurance^3^. In classification or regression applications, a saliency map, also called a sensitivity map or attention heat map demonstrating areas of prominence in determining the result, can be obtained by a variety of methods^4^. However, despite active research in this field, straightforward and clear interpretation of AI models remains a challenge^2^. Currently, most studies employ Grad-CAM^5^ or similar methods. This method is very useful in highlighting the location of an object, such as in a classification task involving a dog in a background, for example. However, it is not satisfactory for interpreting a regression model where most areas in the image contain information reverent for decision making and the model most likely uses a holistic approach. Another simple method to obtain a saliency map is to calculate the gradient, or the partial derivative (PD), of the outcome variable with respect to the intensity of each input image pixel and display this gradient as a heat map^6^. The AI regression model is essentially a highly nonlinear mathematical function that maps multivariable input to an outcome variable. This end-to-end gradient-based saliency mapping could be more appropriate for gaining insights into a regression model. Nonetheless, this approach has been much less commonly employed.

Recently, there have been many reports on using machine learning to determine hand bone age from an X-ray radiograph^7–10^. The hand bone age determination task serves as a good example for investigating saliency mapping. The skeletal maturation of hand bones has been well documented and involves multiple characteristics^11–14^, such as the centers of ossification, size, shape, and texture of individual bones. Traditionally, the determination of hand bone age by radiologists involves determining the age or maturation stage of individual bones and then combining the results to arrive at a final bone age^15^. This process is tedious and suffers from inter- and intra- rater variability. There have been computer software development efforts^16–22^ to automate and assist in hand bone age determination, mostly utilizing features well known to radiologists. Although one would expect a deep learning model to optimally combine all useful information, this has not been demonstrated in an AI model in radiology in general and for hand bone age determination in particular, necessitating further investigation into these models.

Previously, Larson et al.^23^ showed examples of saliency maps for a deep residual network for hand bone age determination. The saliency map was obtained from the absolute value of the PD of the loss function with respect to the intensity of input image pixels and highlighted the importance of bone joints. Ren et al.^24^ demonstrated a regression activation mapping (RAM) tool based on the Grad-CAM method^5^ for a CNN regression model. The weights of features present at the input in the regression stage of the model were used to generate a heat map in the input image space to show important areas for age determination. The heat maps contained a small focal area of high intensity in the age range of 37 to 144 months, suggesting that the model used only a very small area in the radiograph for decision making, a hypothesis needing further testing. In this work, the PD values of inferred bone age with respect to input image intensities were quantified as a saliency marker. This end-to-end gradient-based saliency mapping method has not been previously applied to an AI model for hand bone age determination. The method was applied to a set of test images allowing for statistical analyses.

## Results

### AI model

**Figure 1:**
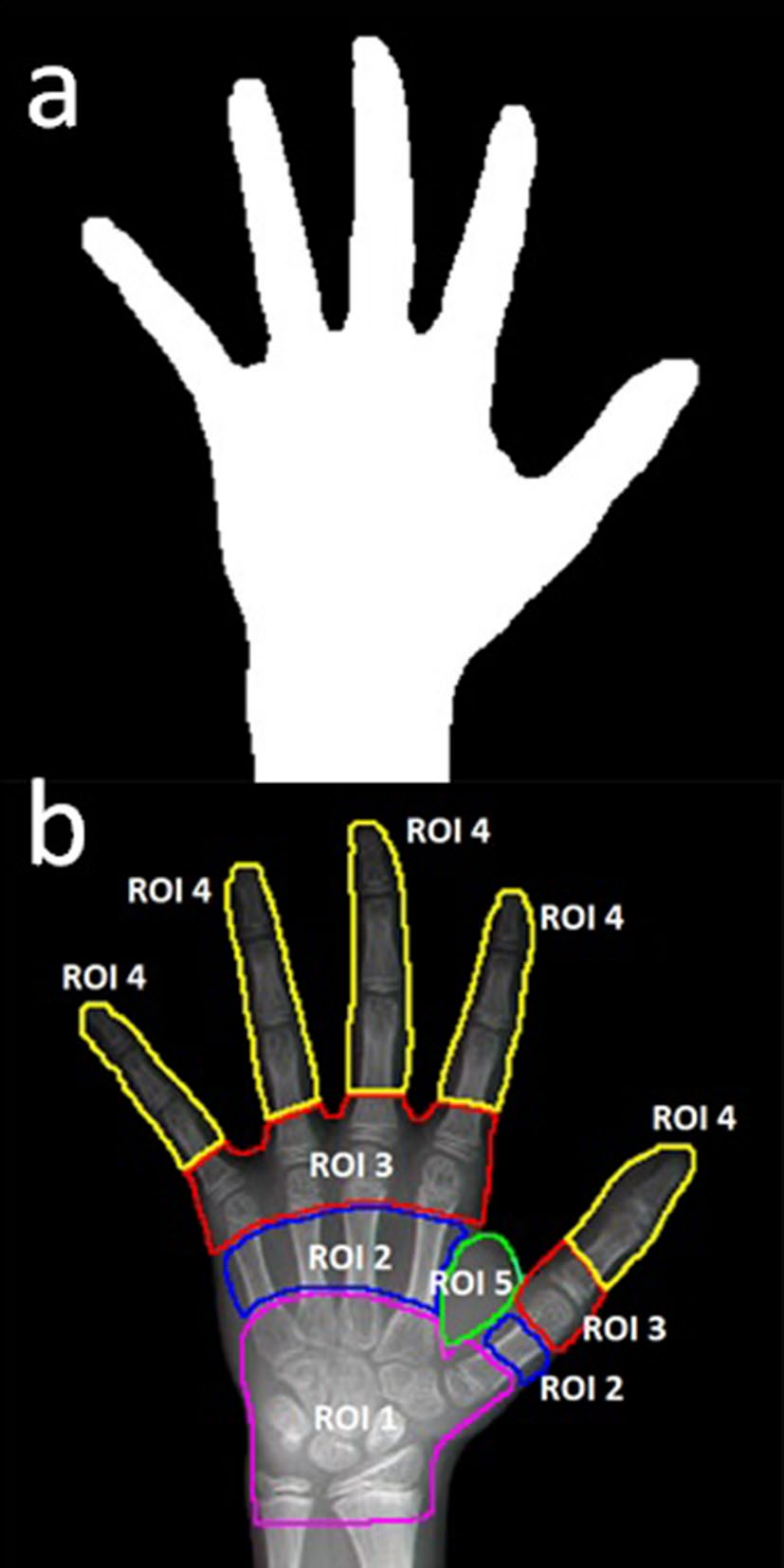
(**a**) A binary mask was generated for all hand images. Outside the hand, the image intensity was set to zero. (**b**) Five ROIs were drawn. ROI 1 covered the wrist area, including carpal bones, the distal ends of the radius and ulna, and the proximal end portions of metacarpal bones; ROI 2 comprised the middle section of the 5 metacarpal bones; ROI 3 comprised the 5 joints connecting metacarpal bones and proximal phalanges; ROI 4 included all areas of phalanges excluding the proximal ends of the proximal phalanges; and ROI 5 was a muscle area between the first and second metacarpal bones containing abductor pollicis, adductor pollicis and flexor pollicis. ROIs 2, 3 and 4 consisted of sub-regions that were not connected.

The hand X-ray radiographs were preprocessed and stored in 3-channel .png files with dimensions of 299 × 299 pixels. The images were multiplied by a binary mask to set areas outside the hand to zero, as shown in Fig. 1a. An InceptionV3 model pretrained with ImageNet was used to map the input image array into 2048 features through global pooling. These features and ages were used to train a regression model with three fully connected dense layers with 1000, 1000 and 100 elements. The testing set contained 100 radiographs with a bone age range of 15 to 216 months (median 150). The mean absolute difference between the predicted age and reference age was 0.68 years, which achieved the accuracy level of typical radiologists^24^.

### PD maps

**Figure 2:**
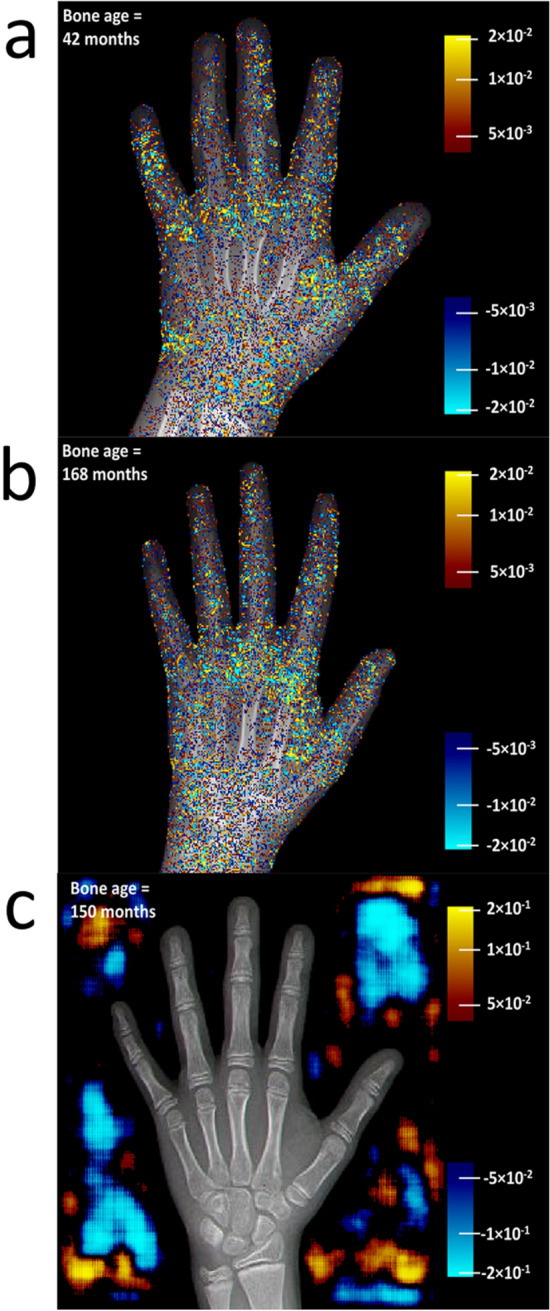
(**a**, **b**) Color-coded PD values were overlaid on the anatomical image. To accommodate a wide range of PD values, the PD values were transformed using a sigmoid function, with ± infinity corresponding to the two extremes on the color scale. The unit of PD scale was months per unit image intensity. The threshold of the color display was set at ± 4 × 10^–3^. (**c**) The PD calculated for the entire image field of view including the area outside the hand mask that was set to zero as input to the AI model in training and inference. Outside the hand mask, the PD values were one order of magnitude larger than inside, and the map had a different type of texture. The threshold of the color display was set at ± 4 × 10^–2^.

The hand and wrist areas were divided into 5 regions of interest (ROIs), as shown in Fig. 1b, for quantitative statistical analyses. The goal of this study was to assess the relative importance of image pixels for determining bone age in each ROI. The PD maps varied among individuals, but consistent patterns across testing images emerged. Figure 2a and b show two examples with different ages. The PDs assumed both positive and negative values and changed rapidly across neighboring pixels. The maps exhibited large amplitudes in the wrist area, especially at younger ages. In addition, the middle section of the metacarpal bones consistently had low values. Surprisingly, the muscle area (ROI 5) also consistently displayed high intensity on the color overlay figures.

The PD maps reveal the pixels of an input image to which the outcome is sensitive. Additional studies were conducted to test PD values outside the hand mask in 5 cases, even though these areas were, by default, set to zero for age inference. The area outside the hand mask had larger PD values than the areas inside the mask (Fig. 2c). This PD map has a different type of texture that is spatially smoother than the texture of other maps.

### Statistical analysis

**Figure 3:**
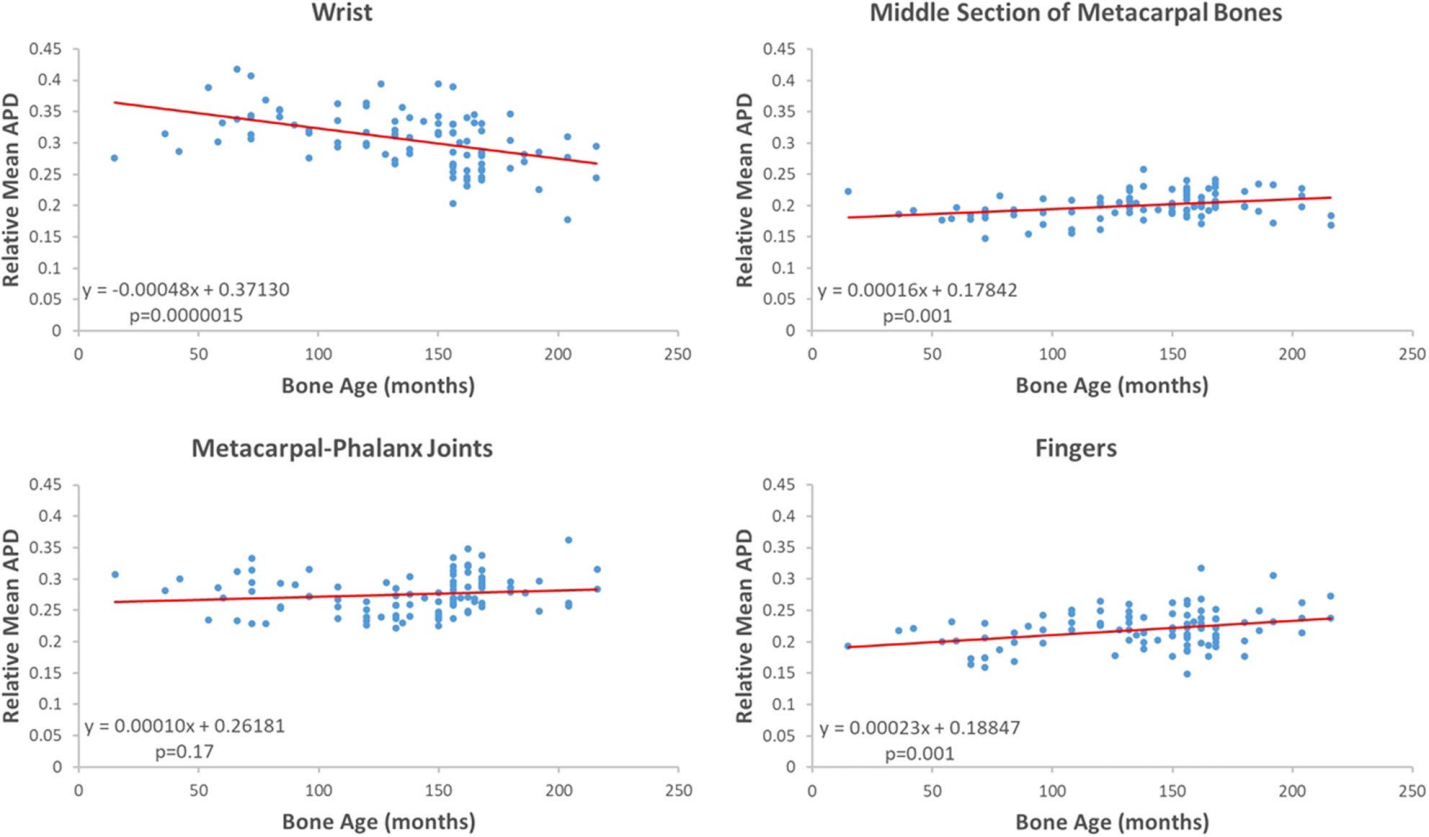
Relative mean APDs were plotted versus the reference bone age for bone-containing ROIs of test data. In each panel, the red line represents the regression equation shown in the figure. The p-value indicates the statistical significance level of the nonzero slope of the regression equation.

Descriptive statistics of the ROI sizes as a percentage of the whole hand mask area are listed in Table 1. Figure 3 shows the scatter plot of the relative mean absolute partial derivative (APD) versus reference bone age for ROIs 1 through 4 as well as the equation of linear regression. Higher APD values suggest more prominence in decision making. A statistically significant nonzero slope was obtained in 3 out of 4 ROIs, indicating that the relative importance of ROIs is age dependent. Surprisingly, the APD values in ROI 5, an area of muscle containing no bone age information, were also high. The linear regression equation for ROI 5 was y = 0.00047x + 0.40166. The statistics of PD values outside the hand mask in 5 cases are also listed in Table 1.

**Table 1:** Statistics of ROI sizes and the APD value for the test data set.

ROI	Anatomical area	Percentage of area of the whole mask (mean ± S.D.)	Average ADP value (in units of months per image intensity unit, mean ± S.D.)
1	Wrist (n = 100)	23.1 ± 1.4%	6.1 × 10^–3^ ± 2.8 × 10^–3^
2	Middle section of metacarpal bones (n = 100)	11.1 ± 1.2%	3.9 × 10^–3^ ± 1.5 × 10^–3^
3	Metacarpal-phalanx joints (n = 100)	20.4 ± 1.1%	5.4 × 10^–3^ ± 2.0 × 10^–3^
4	Fingers (n = 100)	32.7 ± 1.7%	4.3 × 10^–3^ ± 1.7 × 10^–3^
5	Muscles (n = 100)	3.2 ± 0.6%	9.2 × 10^–3^ ± 4.0 × 10^–3^
Outside hand mask (n = 5)		n.a	3.4 × 10^–2^ ± 1.2 × 10^–2^

APD: absolute value of the partial derivative of the predicted age with respect to input image intensity.

## Discussion

In this work, an end-to-end gradient-based saliency map was obtained by calculating the PD of the predicted age with respect to the intensity of individual input image pixels. An increase in image intensity at one pixel affects all features involving this pixel by either increasing or decreasing their amplitude and thus indirectly modify the output from the neural network. Most features extracted by the AI model were presumably fairly localized, which allowed the PD to be used as a marker for saliency. Because neural networks are highly nonlinear, the sensitivity of the outcome to a feature is best studied by assessing how a change in the feature affects the outcome, not the amplitude of the feature itself, making end-to-end PDs particularly useful. The author looked for trends in the absolute PD values and attempted to interpret their behaviors.

The relative importance of ROIs 1 to 4 suggested by Fig. 3 implies that the AI model uses a holistic approach in making the decisions where all ROIs contribute. Based on atlases for hand bone maturation^11–13^, the wrist area undergoes the most drastic changes at a young age, with few carpal bones present at a few months to most bones stabilizing at 8 years of age. This area contains important bone age information^20^. In contrast, the middle section of metacarpal bones is already relatively mature at a very young age and has less predictive value for bone age. In this regard, the AI model appears to examine similar areas used by humans for clues at each age.

Initially, it was surprising to see high PD values in areas that were completely irrelevant to the task, especially the muscular areas between the first and second metacarpal bones (ROI 5). Additional studies showed that PD values outside the hand mask were even higher than values inside. It is known that saliency maps may provide “wrong” information when the feature space contains feathers irrelevant to the task^25^. In our particular model, pixels at the edge of the image field are a part of the feature space. During the training of the model, the network saw only amplitudes of zero for these features. However, the backpropagation training process does not set the weights corresponding to these features to zero. Ultimately, the model has not been trained to handle these features properly and responds to them in an unpredictable manner. In image processing, an AI model may have areas of vulnerability where the outcome is sensitive to perturbation of the input image^26^. Similarly, it can be hypothesized that areas containing irrelevant features may cause errors in the result when unexpected structures or even excessive noise are present. ROI 5 is such an area, containing high PD values but lacking information content regarding the bone age. Small areas with similar properties are expected to exist in bone-containing ROIs (ROIs 1, 2, 3, and 4), although they are not dominant.

The end-to-end gradient-based saliency mapping approach can be readily applied to any black box AI model without knowledge of the network architecture and the weights of the connections. The saliency map obtained with this method can be compared with that obtained by the Grad-CAM method^24,27,28^. The method can be applied to models for which an interpretation may have not been available^29,30^.

There are limitations to this work. Only one AI model was examined. Certain findings of the study may be specific to the model, and the results may not generalize to other models.

## Conclusions

A gradient-based saliency map is useful for providing insights into how a regression AI model makes decisions. The map can be readily calculated by treating the model as a complete black box. When used with other investigation tools, this method may be useful for identifying areas of importance and unimportance for the task.

## Methods

### Data set

X-ray images and bone age from the RNSA pediatric bone age machine learning challenge^8^ were downloaded from Kaggle, and data from males were used. Hands without standard positions for radiography or with malformations were excluded. The remaining images with reference bone age information were randomly divided into training (n = 6021), validation (n = 700) and testing (n = 100) sets.

### Image preparation and AI model

All images were preprocessed in MATLAB to assume an approximately straight orientation with a rear view of the left hand. The image was cropped to fit the hand into a square field of view and resized into an array with dimensions 598 × 598. The background intensity was subtracted from the image. A binary mask was created to set the image intensities outside the hand to zero, as was done by one of the top 5 teams in the RSNA bone age challenge^8^ (Fig. 1a). The mask was generated initially by setting a threshold. Then, an “erode” and “dilate” cycle was applied to remove isolated islands. For a small number of cases, the background in the radiograph was not uniform, and manual tracing was used to remove patches of remaining areas. The 598 × 598 image array was further divided into 299 × 299 cells, each having 2 × 2 pixels, and converted to a 3-channel .png image with 299 × 299 pixels. The button 2 pixels in the cell were copied to the first 2 channels of the image, and the average of the top two pixels was assigned to the third channel. The image was scaled to the intensity range of 0 to 254 as a one-byte unsigned integer array.

The machine learning part of the study was done using Python and Tensor Flow 1.15 and GPU on Google’s Colab cloud computing environment^31^. The training images were randomly augmented with a 10% shift in the up-down direction, 15% shift in the right-left direction, 40 degree rotation, zoom interval [0.90, 1.05], and brightness level interval [0.9, 1.0] to increase the number of instances of training and decrease overfitting. The options used for training included ADAM optimization, a learning rate of 5 × 10^–4^, and MAE monitoring. During training, 100 epochs were run, and the model with the best result on the validation set was saved.

### PD map

A gradient-based saliency map was obtained for each test image. This map could be calculated using backpropagation^1^ but would require knowledge of the weights in the AI model. Instead, the gradient was calculated by using an approximation of the PD of inferred age with respect to the input image intensity of individual pixels. This method is easy to apply even when the neural network is a black box. First, the bone age of the input image is inferred using the AI model described in the previous paragraph. Then, at one pixel of the input image, the intensities of all 3 channels were incremented by one, and the inference of the bone age was repeated. The PD of the predicted age with respect to the pixel intensity was simply approximated by the change in the inferred age since the increment size of the input image intensity was one. For images of the test data set, the mean image intensity within the hand mask ranged from 75 to 124 with a median value of 98. The difference caused by adding 1 to an image pixel was not perceivable to the naked eye, and the change in predicted age was also very small. This procedure was performed for each pixel inside the mask, as exemplified in Fig. 1a. The PD map for each radiograph took approximately 13 min to compute. The PD maps for the testing radiograph set were subjected to statistical analyses.

### Statistical analysis

On each hand radiograph in the testing set, 5 ROIs were defined, as shown in Fig. 1b. The absolute PD values were averaged for each ROI and normalized by the sum of mean values in ROIs 1, 2, 3 and 4 for each radiograph. As a result, for each ROI, we calculated a “relative mean APD”, which was plotted against the reference age. Linear regression was performed to examine the relationship between the relative mean APD and reference age using Microsoft Excel.
